# Understanding and Classifying Metabolite Space and Metabolite-Likeness

**DOI:** 10.1371/journal.pone.0028966

**Published:** 2011-12-14

**Authors:** Julio E. Peironcely, Theo Reijmers, Leon Coulier, Andreas Bender, Thomas Hankemeier

**Affiliations:** 1 TNO Research Group Quality and Safety, Zeist, The Netherlands; 2 Division of Analytical Biosciences, Leiden/Amsterdam Centre for Drug Research, Leiden University, Leiden, The Netherlands; 3 Netherlands Metabolomics Centre, Leiden, The Netherlands; 4 Unilever Centre for Molecular Science Informatics, Department of Chemistry, University of Cambridge, Cambridge, United Kingdom; University of Houston, United States of America

## Abstract

While the entirety of ‘Chemical Space’ is huge (and assumed to contain between 10^63^ and 10^200^ ‘small molecules’), distinct subsets of this space can nonetheless be defined according to certain structural parameters. An example of such a subspace is the chemical space spanned by endogenous metabolites, defined as ‘naturally occurring’ products of an organisms' metabolism. In order to understand this part of chemical space in more detail, we analyzed the chemical space populated by human metabolites in two ways. Firstly, in order to understand metabolite space better, we performed Principal Component Analysis (PCA), hierarchical clustering and scaffold analysis of metabolites and non-metabolites in order to analyze which chemical features are characteristic for both classes of compounds. Here we found that heteroatom (both oxygen and nitrogen) content, as well as the presence of particular ring systems was able to distinguish both groups of compounds. Secondly, we established which molecular descriptors and classifiers are capable of distinguishing metabolites from non-metabolites, by assigning a ‘metabolite-likeness’ score. It was found that the combination of MDL Public Keys and Random Forest exhibited best overall classification performance with an AUC value of 99.13%, a specificity of 99.84% and a selectivity of 88.79%. This performance is slightly better than previous classifiers; and interestingly we found that drugs occupy two distinct areas of metabolite-likeness, the one being more ‘synthetic’ and the other being more ‘metabolite-like’. Also, on a truly prospective dataset of 457 compounds, 95.84% correct classification was achieved. Overall, we are confident that we contributed to the tasks of classifying metabolites, as well as to understanding metabolite chemical space better. This knowledge can now be used in the development of new drugs that need to resemble metabolites, and in our work particularly for assessing the metabolite-likeness of candidate molecules during metabolite identification in the metabolomics field.

## Introduction

The area of ‘Metabolomics’ is relatively young [1], [2] and describes the large-scale analysis of (often human and endogenous) metabolites. It comprises both the analytical approaches employed, such as mass spectroscopy (MS) as well as the analysis of the resulting data on a network- and phenotype level. Metabolomics is a particularly interesting research field as it allows the determination of biological phenotypes on a chemical basis, since endogenous metabolites are closer phenotype of an organism than for example gene expression [3]. As a consequence, new knowledge on biological processes can be obtained by investigating metabolites.

Various experimental techniques, most commonly MS and nuclear magnetic resonance (NMR), have been devised to detect and identify metabolites, with different approaches being necessary to cover different parts of the metabolite spectrum. In practice it is found that some metabolites with different lipophilicity can only be detected by one of the experimental techniques but not by others [4]–[9]. Different techniques might also be used depending on the type and quantity of sample to be analyzed, as well as the concentration and the molecular properties of the metabolites. In general terms, NMR allows for a detailed characterization of the chemical structure of the (un)known compound, and it is the preferred technique for unambiguous identification of a chemical structure. On the downside, NMR requires abundant and pure sample, yielding low sensitivity. Conversely, MS offers high sensitivity and specificity, requiring less amounts of sample, but providing less information about the chemical structure, namely its elemental composition and some structural fragments.

However, despite its ability to describe a phenotype in many cases in a more relevant manner than other approaches, in metabolomics studies a major challenge exists, namely metabolite identification [10]–[12]. While many endogenous metabolites can be detected (and their spectrum determined), also elucidating their chemical structures is essential to properly interpret results, and to utilize the analytical data to finally answer biological questions [13]. However, the step from the analytical readout to the structural formula is often fraught with problems.

In the commonly employed MS-based profiling approaches (which are also used in our group), once metabolites are detected their elemental composition (or multiple elemental compositions) [14], [15] can be derived directly from MS data. Based on this elemental composition, matching chemical structures can be proposed following two approaches. In the first approach, molecular databases are queried for the presence of molecules with the same elemental composition (or similar spectral data), and hits are returned as candidate structures [13], [16]. However, the major shortcoming of this approach is that one can only find in databases what has been found before, making the elucidation of novel metabolites impossible. In the second approach, which is meant to cover this shortcoming, the elemental composition and optionally other experimental data are provided to a ‘structure elucidator’, which will generate *in silico* all possible chemical structures which match the analytical constraints provided to the algorithm [17]–[19]. While one of the structures generated will be the metabolite of interest, depending on the elemental formula provided, the latter method in particular yields a large number of possible solutions. (For example, the elemental composition of phenylalanine, C9H11NO2, yields 277,810,163 possible candidate structures.)

Due to the above reasons, molecular databases compiling structural information on endogenous metabolites are currently limited in size and they certainly do not cover metabolite space exhaustively. The number of possible metabolites is yet unknown [20]. While lipids alone are estimated to exist in the order of 20,000 different structures [21] plants are thought to contain around 200,000 metabolites [3]. Given these figures, the experimental data obtained until today is relatively scarce. A large database of metabolites such as the Human Metabolome Database (HMDB) [21] contains in its current version about 8,000 structures, which is only a fraction of the above numbers. Still, HMDB is the most comprehensive dataset to represent the Metabolite Space from a human point of view. Plant metabolomics makes use of different databases [12]. In addition metabolomics databases exist [22] that contain metabolites and the enzymatic reactions that connect them to pathways, such as in KEGG [23]; some databases contain metabolites grouped by organism such as in BioCyc [24] and other database relate metabolites with experimental information, such as Metlin [25]. Still, given its number of data entries, the approach to match the MS or NMR spectrum to database spectra can only succeed in a fraction of cases.

Hence, solutions need to be ranked, based on the likelihood of a molecular structure to be a metabolite [26] – and, as we will outline in more detail below, this is one of the main aims of the current work of implementing a ‘metabolite-likeness’ model. In addition, our goal was to understand metabolites better from a chemical point of view, and this is what we will discuss in the remainder of this work, after setting our approach in context with the ‘prior art’ in the field of metabolite classification.

Focusing on metabolites of *E. coli*, Nobeli et al. [27] studied 745 metabolites of this organism by analyzing physiochemical descriptors, the diversity of scaffolds, and similarity-based compound clustering. It was observed that most of the *E. coli* metabolites are found between the 100 and 300 Da molecular weight region, that they contain up to 20 heavy atoms, and that they are mostly hydrophilic. In addition the low diversity of molecular scaffolds was observed. The clustering analysis performed revealed that it is difficult to use molecular similarity to group metabolites in ‘sub-classes’, since there is not a natural separation according to their two-dimensional structure similarity, concluding that the metabolite space of *E. coli* is homogeneous.

While Nobeli et al. focused on the metabolome of *E. Coli*, Gupta et al. [28] represented the chemical space of metabolites using the KEGG/LIGAND database, which includes metabolites from different species as well as xenobiotics. The chemical space of non-metabolites was approximated by ZINC database [29], which contains small molecules that are commercially available. These molecules are often used as the search space, in virtual-screening research, or as background set in classification projects.

In this work it was concluded that hydroxyl groups, aromatic systems, and molecular weight are discriminating features between metabolite and non-metabolite chemical space. Furthermore, Self Organizing Maps (SOM), Random Forests (RF), and Classification Trees (CT) were employed to distinguish between the two classes of compounds, which were represented by 3D descriptors, topological descriptors, and global molecular descriptors, respectively. The best classification accuracy was 97%, achieved by the combination of RF and global molecular descriptors. (No external validation of such models is reported in their work, as opposed to our novel study, which includes a prospective validation set.)

While trying to discriminate metabolites from non-metabolites was the obvious starting point, it was then noted that also bioactive compounds, notably drugs, could be related to the metabolite/non-metabolite chemical spaces. All three of those sets were hence analyzed by Dobson et al. in a subsequent study [30]. Endogenous metabolites were selected from the HMDB, BioCyc, BiGG, and Edinburgh databases while drugs were compiled from DrugBank and KEGG DRUG. In addition, screening molecules from ZINC were the source for the background compound set. Molecules were represented using connectivity and path fingerprints, MDL Public Keys and E-state, and the similarity between them was determined by the Tanimoto coefficient. In this work the authors concluded that *drugs are more similar to metabolites than to screening compounds*. Furthermore the distribution of molecular properties among the different families of compounds was studied and it was noticed that metabolites tend to have fewer heavy atoms than the other two groups of compounds. Another relevant physicochemical property identified was lipophilicity, which showed a bias in metabolites towards hydrophilicity, whereas drugs and screening compounds were more hydrophobic.

In the current study we are extending previous work by, compared to Gupta et al., focusing on a large set of human metabolites obtained from HMDB, instead of metabolites from multiple species, and an updated collection of background compounds from ZINC. We make use of different molecular descriptors such as ECFP_4 [31], FCFP_4, MDL Public Keys [32], and physicochemical properties, as well as classifiers like Support Vector Machines (SVM) [33], Random Forest (RF) [34] and Naïve Bayes (NB) [35] and evaluate their applicability to distinguishing metabolites from non-metabolites. In addition we include a prospective validation set to further assess model performance. Furthermore, Dobson et al. used molecular similarity to metabolites as an indicator of metabolite-likeness. In comparison, we assign our score based on the predictions given by different classification methods. The classifier presented here employs, at the time of publication, the most comprehensive collection of human metabolites and purchasable compounds. Furthermore we also make use of PCA and hierarchical clustering to understand which physicochemical properties as well as chemical functionalities are characteristic of metabolites, and discriminate them from non-metabolites. The principal aim of this work is to establish a reliable metabolite classifier for candidate structures that need to be identified in metabolomics studies; however, apart from the classifier itself, also understanding metabolite space better was a second major aim of this work.

## Methods

### Datasets and Data Preprocessing

The Human Metabolome Database (HMDB) version 2.5 [21] served as source of the metabolite set. This database contains, in its original form, 7,886 human metabolites as determined by experimental analytical methods. The ZINC Database (ZINC) release 8 [29] was chosen to represent non-metabolite chemical space. From the different datasets provided by ZINC, we selected the subset “everything #10” (date 2010-06-17), since it includes 21.6 million compounds and it was the largest set at the time, and, hence, most representative of ‘all’ chemical space.

Molecules from the two datasets were standardized with PipelinePilot Student Edition 6.1 [36] using the ‘washing’ workflow suggested by Dobson et al. [30], which involved the selection of the largest fragment in the structure, the removal of salts and hydrogen atoms and the standardization of charges and stereochemistry. Because the ZINC database mainly contains molecules with a low molecular weight, a value of 1000 Daltons was set as the maximum molecular weight of any compound, metabolite or not, in this study. While this removes part of chemical space from the metabolite dataset, this step was necessary to avoid molecular weight to appear as a major discriminant between metabolites and non-metabolites (which would not be relevant in the context of our future application of distinguishing metabolites from non-metabolites in cases of structures with an identical sum formula). Furthermore, when employing fingerprints for classification, the chemical distribution of features (as opposed to the molecular weight) will be used for classification, hence making the classification (in this feature space) size-independent. This filter removed 775 metabolites from the HMDB dataset. Furthermore, the constraint imposed on molecules to contain three or more atoms (in order to retain only small organic molecules in the dataset) removed 65 small molecules and ions from HMDB. Metabolites from HMDB that are considered drugs were also removed from the dataset, based on annotations as drugs in the fields “Taxonomy Family” and “Taxonomy Sub Class” provided by HMDB, removing 92 drugs from the dataset and reducing the metabolite dataset to 6,954 molecules. The number of molecules contained in ZINC was excessively large to perform clustering and classification, concerning the computational resources needed for such tasks, therefore selecting a subset was necessary. Such a subset was randomly selected from ZINC, which contained 194,350 molecules. All of these molecules passed the filtering based on molecular weight and the minimum number of atoms. The last dataset preprocessing step was the removal of metabolites (molecules contained in the HMDB database) from the ZINC dataset, where 8 molecules were removed from the non-metabolite set.

### Training and Test Sets

Diversity selection [37], [38] was used in this work to prepare representative compound datasets for metabolites and non-metabolites with the intention of reducing the bias that overrepresented families of molecules could have on the classification step. This initially appeared particularly crucial since lipids were hugely overrepresented in the HMDB database. After giving it more thought it was noted that this step certainly involves subjective elements since it, on the one hand, removes information about the distribution of data points in the original set. On the other hand, we assumed that there was a significant bias present in particular in the metabolite dataset not only due to ‘natural’ causes, but also due to the bias introduced by experimental techniques (such as MS and NMR), which are able to detect and identify compounds rather selectively. Hence, we came to the conclusion that close analogues should be removed carefully from the dataset. In this spirit, each dataset was independently clustered using the maximal dissimilarity partitioning algorithm implementation from the ‘Cluster Molecules’ component from PipelinePilot Student Edition 6.1 [36]. Molecules were represented by ECFP_4 fingerprints and the distance between each pair of molecules was calculated using the Tanimoto coefficient. The maximum dissimilarity of a cluster member to the cluster centre was 0.6, (that is, molecules from the same cluster possess a ECFP_4/Tanimoto similarity of at least 0.4). Finally cluster centers were selected as representatives of each cluster, which yielded 532 representatives for HMDB and more than 12,000 for ZINC. In order to have balanced training datasets for model building (where some algorithms are prone to majority class predictions), 532 random molecules were selected from ZINC. These two subsets of 532 molecules each were used for building the classification models. While these datasets are small, they were intended to remove much of the bias present in the original datasets. We also still made use of the additional compound information available since from the remaining molecules not included in the training datasets the test set was built, where the remaining 6,422 metabolites as well as 6,422 randomly selected non-metabolites were joined to form an initial test set of 12,844 molecules. Hence, this very large test set was used to evaluate whether model generation with our training dataset assembled in the way just described would produce viable metabolite-likeness models.

### Prospective Validation Sets

Predictive models are meant to be applied to novel, unseen molecules, and to estimate the performance on those new molecules the utilization of external validation sets is crucial. In order to determine prospective performance of our model, an external validation set was compiled, which includes 563 metabolites not yet part of HMDB (which were provided by the database curators). After filtering using the standardization protocol described above, the resulting prospective validation set contained 457 metabolites that were not included in any of the previous preprocessing steps (diversity selection, model building, and model evaluation). Furthermore, two other datasets of molecules were assembled for evaluation with the metabolite-likeness model, namely one of drugs, and one of bioactive compounds (as determined by experimental assays). To represent drugs DrugBank release 2.5 (date 23-11-2010) [39] was used, comprising 6,532 molecules. To represent bioactive molecules, ChEMBL [40] release 8 (date 09-12-2010) was employed. Both datasets were normalized using the protocol described above and from the 635,933 compounds in ChEMBL, 6,312 were randomly selected (the DrugBank dataset was used in full due to its smaller size). With these datasets we evaluated if our metabolite-likeness model is able to detect the biogenic bias of drugs and bioactive compounds in general. With these three prospective validation sets (external validation set, drug set, bioactive compound set) we evaluated our best model, as derived in the parameter exploration, in two different ways. Firstly, the quality of the predictions for metabolites that were not involved at any stage of the model creation by employing an external validation set, was determined. Secondly, we tested the hypothesis that drugs (and, possibly to a lesser extent, bioactive molecules) are more similar to metabolites than to non-metabolites. This hypothesis could either be rejected or not from the distribution of metabolite-likeness scores as assigned by our model.

### Molecular Descriptors

Molecular descriptors should be chosen with care depending for which problem they are going to be used [41], [42]. In this case different descriptor sets were used for classification as follows.

#### a) Atom Counts and Physicochemical Molecular Descriptors

Atom counts and physicochemical descriptors are rather simple, intuitive and easy to interpret by chemists. On the downside, they usually result in poorer classification results than more complex descriptors since no structural information is captured. In this study our descriptor set based on atom counts was called ‘Atom Counts’ and contained counts of the most common atom types in metabolites, namely H_Count, C_Count, N_Count, O_Count, F_Count, P_Count, S_Count, Cl_Count. ‘Atom Counts’ descriptors were computed using the component ‘Element Count’ from PipelinePilot Student Edition 6.1 [36]. The physicochemical properties used were the Atom Counts descriptors mentioned above together with the following properties: the number of atoms (Num_Atoms in PipelinePilot), a calculated logP value (ALogP), a calculated logD value (LogD), the number of hydrogen donors (Num_H_Donors) and acceptors (Num_H_Acceptors), the number of rotatable bonds (Num_RotatableBonds), the number of rings (Num_Rings), the number of aromatic rings (Num_AromaticRings), a calculated value of solubility (Molecular_Solubility), a calculated value of the polar surface area (Molecular_PolarSurfaceArea), and a calculated value for the minimized energy (Minimized_Energy). All these properties, listed in detail in Table 1, were calculated with the components ‘Element Count’, ‘Calculate Properties’, ‘ALogP’, ‘LogD’, ‘Surface Area and Volume’, ‘Molecular Energy’ as implemented in PipelinePilot Student Edition 6.1 [36].

**Table 1:** List of atom counts and physicochemical properties used to describe the molecules of this study.

Descriptors	Properties
Atom Counts	H_Count, C_Count, N_Count, O_Count, F_Count, P_Count, S_Count, Cl_Count
PP_desc	Atom Counts, Molecular_Weight, Num_Atoms, ALogP, LogD, Num_H_Donors, Num_H_Acceptors, Num_RotatableBonds, Num_Rings, Num_AromaticRings, Molecular_Solubility, Molecular_PolarSurfaceArea, Minimized_Energy

PP_desc include Atom Counts and the listed physicochemical properties.

#### b) Fingerprints

2D ECFP_X and FCFP_X are “Extended Connectivity” molecular fingerprints where features are descriptions of the neighborhood of the atoms up to a certain distance or radius X. In the ECFP fingerprint the atom identifier is based on the atom type, while in FCFP it is based on the functional class of the atom [31]. In this work, ECFP and FCFP fingerprints with radius 4 were calculated using the component ‘Molecular Properties’ in PipelinePilot Student Edition 6.1 [36] with the parameter ‘Convert Fingerprint To’ set to ‘Leave As-Is’. These fingerprints can produce thousands of features for a molecular library, including features that are present in very few molecules, which can easily lead to over fitting. Hence, we folded the fingerprints to a fixed length of 1024 bits, using PipelinePilot Student Edition 6.1 [36] component ‘Convert Fingerprint’, to an output format of ‘Fixed length Array of Bits’, ‘Fixed Bit Length’ of 1024, and ‘Output Bit Order’ of ‘Pack Least-Significant First’.

MDL keys [32] were used as well for classification. MDL Public Keys are a key-based molecular representation defined by the presence or absence of 166 predefined keys, or molecular substructures. Since the size of this key set is only 166 bits, folding is not necessary.

### Principal Component Analysis

Principal Component Analysis (PCA) is a mathematical transformation that projects the dataset onto a lower dimension defined by uncorrelated variables, the so-called ‘principal components’ [43]. Such components are ordered according to the percentage of variance in the dataset that they explain, which means that the first principal component explains the highest variance. We performed a PCA on the training set of metabolites and non-metabolites in order to understand better the nature of the chemistry contained in both classes. PCA was performed using the R library *FactoMineR* [44] and data was standardized to unit variance before analysis.

### Hierarchical Clustering

Hierarchical Clustering groups objects together that are close in the particular representation chosen and assigns a hierarchy to the resulting clusters. This grouping can be agglomerative, where initially each object is a cluster by itself and where clusters are subsequently combined, or divisive, where the whole dataset is assigned to a single cluster initially which is then iteratively split into smaller clusters. Furthermore, two other factors determine the output of the clustering, the distance metric between objects and the method used to link two clusters, *i.e.* the method used to calculate the distance between clusters. We have used the agglomerative hierarchical clustering offered by *FactoMineR* [44] on the results of the PCA as described above in combination with an Euclidean distance metric and Ward's linkage method. Finally, the hierarchy of clusters is presented on a dendogram that needs to be cut at some point to split the clusters. The criteria employed to cut the dendogram was the default in *FactoMineR*, which splits the clusters at the point of maximal loss of intra-cluster inertia. The clustering results are used to evaluate if some natural grouping emerges from the data; in our case, whether metabolite space actually contains several distinct subspaces.

### Classification Trees

Classification trees are machine-learning methods that use a univariate partition to split the dataset in subsets [45]. At each step the data is split using the predicting variable that optimizes a certain criteria. In our case, we make use of conditional inference trees (CIT) as implemented in the R package *party* [46]. Conditional inference trees perform a covariate selection that relies on permutation tests and statistical significance. Applying CIT to a two-class classification problem can be seen as a binary tree where at each node the dataset is split into two subsets using the covariate that has the strongest association to the response variable. In the case that features are binary fingerprints, the presence or absence of a given feature determines the data split performed. Variables are selected if they maximize the ‘purity’ of the split, this is, that each subset contains mostly objects of one class. The result is a tree that depicts the best variables to split the data and provides information about relevant variables for each class of objects. In the course of the present study, classification trees were applied particularly to ECFP_4 fingerprints, in order to determine which features distinguish metabolite space from non-metabolite space.

### Fragment Analysis

In this part of the work, we further analyzed the fragment composition of metabolite and ‘purchasable chemistry’ spaces as a means to better understand the composition of (and differences between) both compound spaces. From the point of view of a chemist, molecular fragments are easier to interpret and convey more meaning than a fingerprint or a sensitivity percentage. Therefore we used the component ‘Generate Fragments’ from PipelinePilot Student Edition 6.1 [36] to enumerate (in PipelinePilot terminology) rings, ring assemblies, bridge assemblies, chains, and Murcko assemblies (scaffolds that contain ring systems and ring systems connected by linkers, but no side chains) [47]. The top 20 most frequent fragments from our two datasets, human metabolites and purchasable compounds were collected and analyzed.

### Machine Learning

Three machine-learning algorithms were used to generate the models of metabolite-likeness, namely Support Vector Machines (SVM) [33], Random Forests (RF) [34], and the Naïve Bayes Classifier (NB) [48]. We used the implementations of these algorithms in the statistical software package R [49]. For SVM, we employed the library *e1071* [50], which is an implementation of the standard C++ *libsvm* [51]. As for RF, we opted for the library *randomForest* [52], an R port of the original code of Breiman [34]. Again *e1071* was the library chosen for NB.

SVM is one of the most robust and widely used algorithms in machine learning and it belongs to the class of maximum margin classifiers [33], [53]. In a two-class problem, SVM tries to define a boundary that maximizes the separation between the two classes. Provided the classes are linearly separable, SVM builds a hyperplane with a maximal margin to neighboring objects of the two classes. When the linear separation is not feasible, a kernel function executes a nonlinear mapping of the data to a higher dimension where it can be linearly separated. SVM requires the tuning of two metaparameters, gamma, which regulates the level of non-linear behavior of the kernel, and *C*, the cost of violating the constraints, in order to achieve an optimal performance. The kernel type was set to the default Gaussian Radial Basis Function (RBF). SVMs have been successfully used in molecular classification before, such as for classifying ‘drug-likeness’ [54], [55].

RF is an ensemble of classification trees [34] in which each tree classifies, or votes, the class of an object given a randomly chosen subset of the full variable set. Many of such trees are grown (as determined by the variable ntree) and majority voting is used to obtain one final classification result. RF requires the tuning of the metaparameter mtry, which determines the number of variables randomly sampled.

The last classification algorithm is the Naïve Bayes algorithm [48], which relies on the assumption that the variable values are conditionally independent of the class label. This strong assumption usually does not hold, but in practice this approach still allows building good models for multidimensional data, as was shown for bioactivity datasets before [56], [57]. Compared to SVM and RF, NB only requires one parameter to be tuned, the cut-off value for the class membership probability (equivalent to changing the choice of the ‘prior’), which was however not explored in this work and it was set to its theoretical optimum (it was set to 50% in the case of balanced datasets, as proposed previously) [58]. According to this, a molecule with a predicted metabolite-likeness of 50% of higher is considered to be a metabolite, and with less than 50% metabolite-likeness, a non-metabolite.

### Cross Validation and Model Generation

Concerning RF and SVM, k-fold cross validation [59]–[61] is a recommended method to tune metaparameters and avoid over fitting. We opted to apply a 5 fold cross validation, a previously recommended value for k [62], [63], to the 1,064 molecules in the training dataset. In the case of RF, for each cross validation split a range of values for mtry metaparameter were tested, while the number of trees in the forest, ntree, was set to the default value of 500. The mtry giving the highest averaged Area Under the Curve (AUC) and smallest classification error was chosen as the optimal value for building the model. Cross validation was performed in the same fashion for SVM (Table S1 shows the best values obtained for the metaparameters). Once the optimal metaparameters were selected, final RF (RF variable importance of PP_desc descriptors are listed in Table S2, and for MDL Public Keys in Table S3), SVM, and NB models were generated using the complete set of 1,064 molecules in the training dataset. This process of metaparameter determination and model building was performed for each pair of three different classifiers (RF, SVM, and NB) and five molecular representations (PP_desc, Atom Counts, ECFP_4, FCFP_4, and MDL Public Keys), resulting in a total of 15 different classification exercises.

### Model Benchmarking

Once the training step was finished, we needed to evaluate what pair of classifier and representation gave the best results on the test set, consisting of an additional 6,422 metabolites as well as 6,422 non-metabolites that were not used at any stage during model training. To evaluate model performance we used sensitivity and specificity values derived from the confusion matrices, together with ROC curves and their associated AUC. After applying the models to the test set, the final step involved classification of the molecules contained to the prospective, external validation sets described above. The distribution of the metabolite-likeness scores for these datasets as well as the percentage of correctly classified compounds are discussed in the Results and Discussion section.

## Results and Discussion

### PCA and Hierarchical Clustering

**Figure 1:**
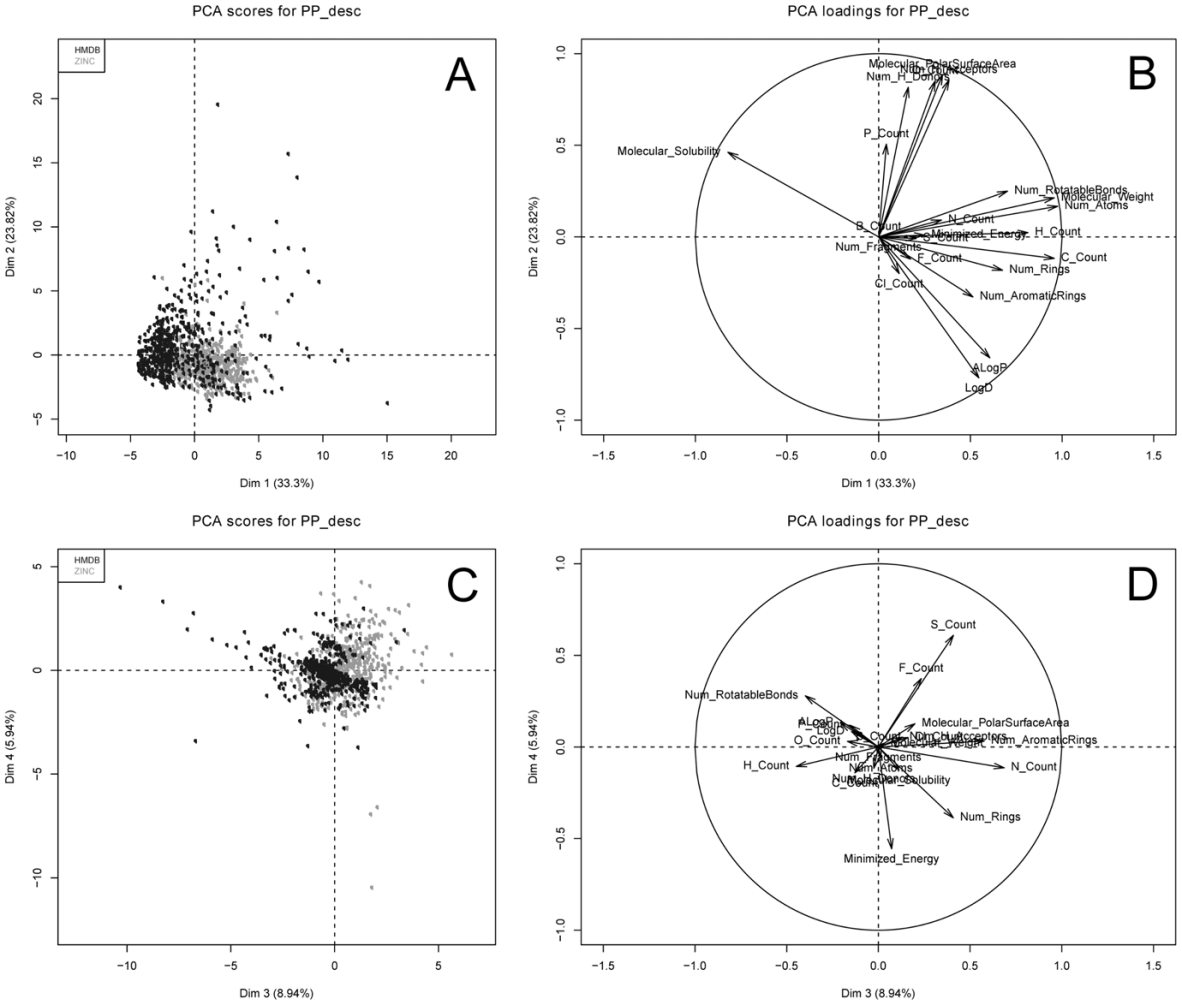
Principal Components Analysis of the PP_desc training set. PCA plots (A,C) and variable contributions(B,D) for the training datasets PP_desc.

PCA was performed to the training set and the loadings and scores plots for the first four dimensions are presented in Figure 1. For this PCA, we focus on physicochemical properties (PP_desc) for the sake of interpretability (PCA results for MDL Public Keys are presented in Figure S1 and Figure S2, and the percentage of variance explained in Table S4). Almost 71% of the variance is explained in the first four components. A slight separation between metabolites and non-metabolites can be observed in the score plots of PP_desc (Figure 1A and Figure 1C). The loadings plots for PP_desc (Figure 1B and Figure 1D) one can see which variables are correlated or inversely correlated with each class of compounds. For the first two dimensions (Figure 1B), the variables that contribute the most to the variance are Molecular Solubility, Molecular Weigh, Molecular Polar Surface Area (PSA), and the number of carbon atoms per molecule (C_Count). Metabolites hence tend to have higher water solubility, lower molecular weight, and fewer carbon atoms than non-metabolites. These observations are in accordance to the work of Nobeli et al. [27] and Dobson et al. [30], who concluded that metabolites are hydrophilic and have less heavy atoms than non-metabolites. PSA tends to be bigger than the one of non-metabolites, suggesting that metabolites do not penetrate cell membranes as efficiently as the non-metabolites. Furthermore, the loadings plot for the third and fourth dimensions (Figure 1D), shows that the most contributing variables are Num Rings, Num Rotatable Bonds, N Count, S Count, and Minimized Energy. The number of rings, rotatable bonds, and minimized energy, for which metabolites obtain lower values than non-metabolites, are indicators of molecular complexity, and, therefore, one can conclude that metabolites have simpler chemical structures than non-metabolites. Interestingly, metabolites also have fewer nitrogen and sulfur atoms than non-metabolites, as is the case for all atom types except for oxygen and phosphor, which are more frequent for metabolites as opposed to non-metabolites.

The results of the PCA of PP_desc and MDL Public Keys were subject to hierarchical clustering. (Plots are presented in Figure S3.) In both cases the optimal cluster split, according to the loss of intra-cluster inertia, returned 3 clusters. The distribution of metabolites and non-metabolites in each cluster is listed in Table 2. It can be seen that for PP_desc and MDL Public Keys 2 large clusters and a third small one are formed, each of them containing one dominant class of compounds. The first cluster for PP_desc has a purity of 70.2% (370 metabolites and 157 non-metabolites), the second cluster has a purity of 89.65% (52 metabolites and 6 non-metabolites), and the third cluster has a purity of 77.03% (110 metabolites and 369 non-metabolites). Using MDL Public Keys, the first cluster has a purity of 78.81% (372 metabolites and 100 non-metabolites), the second cluster has a purity of 73.03% (134 metabolites and 363 non-metabolites), and the third cluster has a purity of 72.63% (26 metabolites and 69 non-metabolites). However, the purity of each cluster is not high and this, together with the lack of separation observed in the PCA, leads us to think that the separation of metabolites from non-metabolites requires the utilization of more sophisticated methods like random forests, or other nonlinear classifiers as explored in the following.

**Table 2:** Cluster distribution of the molecules in the training datasets, using PP_desc and MDL Public Keys.

Cluster	Type	PP_desc	MDL Public Keys
1	HMDB	370	372
1	ZINC	157	100
2	HMDB	52	134
2	ZINC	6	363
3	HMDB	110	26
3	ZINC	369	69

The clustering performed was a hierarchical clustering and the dendogram was cut at the point of maximal inertia loss.

### Fingerprint Features and Fragment Analysis

**Figure 2:**
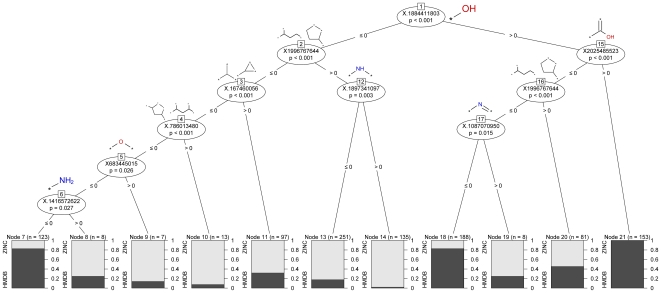
Conditional inference tree of the ECFP_4 features in the training set. Hydroxyls, carboxylic acids, and linear structures are associated with metabolites, whereas secondary amines and secondary imines are associated with non-metabolites.

**Figure 3:**
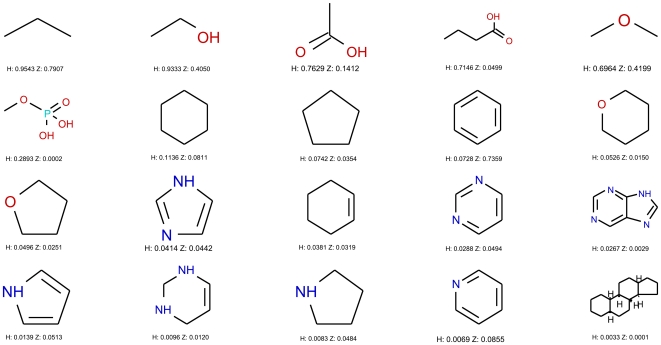
Top 20 most frequent fragments in HMDB. The 20 most frequent ring systems, chain assemblies, and Murcko assemblies in the metabolite data set (HMDB compounds). ‘H’ refers to the frequency of fragments in the HMDB dataset, ‘Z’ to the frequency of fragments in the ZINC dataset. Fragments with less than 4 heavy atoms were excluded. Oxygen containing rings, phosphate group, hydroxyl, carboxylic acid, and the steroid scaffold, among others, are common fragments in metabolites.

A classification tree was built upon the training set, which was described using non-hashed ECFP_4 fingerprints (Figure 2). The results give a general idea of which chemical moieties are characteristic of each class of compounds. As expected, the most discriminating feature was the hydroxyl group, in agreement with the work by Gupta et al. [28], with a higher frequency among metabolites. On the other hand, the presence of chemical moieties containing nitrogen, in particular secondary amines and secondary imines, is highly correlated with a class membership of the non-metabolites. Finally, in the case a molecule lacks hydroxyl functionalities (demonstrated to be metabolite-like moieties), but it also lacks five or three member rings, ether-like features, and primary amines, it will likely be a metabolite (which is the combination of features in the left-most branch of the tree in Figure 3).

**Figure 4:**
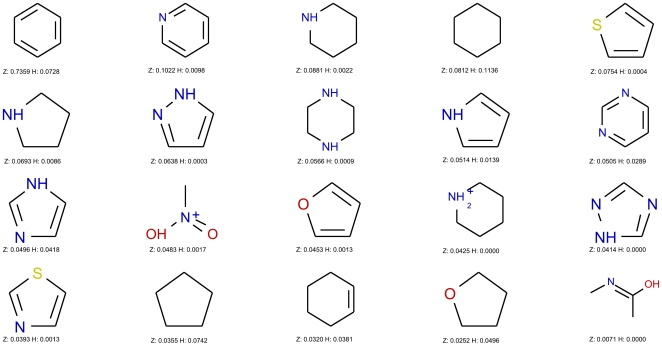
Top 20 most frequent fragments in ZINC. The 20 most frequent ring systems, chain assemblies, and Murcko assemblies among the ZINC compounds, here chosen as a non-metabolite-like set. ‘H’ refers to the frequency of fragments in the HMDB dataset, ‘Z’ to the frequency of fragments in the ZINC dataset. Fragments with less than 4 heavy atoms were excluded. Nitrogen containing rings dominate the most frequent fragments.

When looking at the frequent fragments of metabolites (Figure 3) and non-metabolites (Figure 4), we corroborate this finding. Among metabolites, hydroxyls and carboxylic acids are frequent as well as rings containing oxygen atoms. In the case of non-metabolites, either rings or linear fragments containing nitrogen and sulfur abound, which is in accordance to the classification tree results, in accordance to the findings of Hert et al. [64]. Other frequent fragments of metabolites are the phosphate group, characteristic of some classes of metabolites like nucleotides and phospholipids, as well as the steroid and adenine scaffolds. This importance of class-specific fragments can make two metabolites from different classes very different, and it hence poses a challenge when building models that aim to capture such diversity within a given class. One option is to build local models for each subclass of metabolites; but in this study we aimed at building a global model for metabolites, and as a result, we rely on complex classifiers to predict the metabolite-likeness of molecules. These classification models were built using the methods and data described in the methods section and they were applied to our test set.

### Test Set

**Figure 5:**
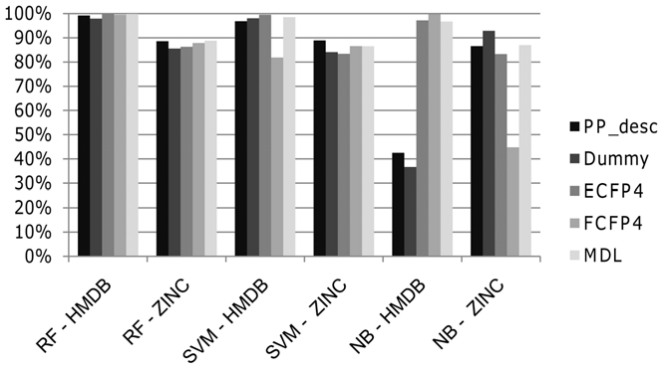
Classification accuracy on the test set. Percentage of correctly classified molecules of the test set for each combination of fingerprint and classifier. Sensitivity is in most cases larger than 90%, except for FCFP_4 and SVM, and Atom Counts and PP_desc and NB. Specificity is larger than 80% in most cases, except FCFP_4 and NB. It can be observed that metabolites are classified more accurately than non-metabolites when using RF and SVM.

In this study we used 5 molecular representations and 3 classifiers. Our aim was to select which combination of molecular representation and classifier yielded the best classification results for metabolites. The classification results on the test set for each combination are presented in Table 3 and visualized graphically in Figure 5. MDL Public Keys and RF, reporting 99.84% sensitivity and 88.79% specificity, achieve best results. ECFP_4 is the best performing molecular representation when used with SVM, achieving 99.55% sensitivity, while PP_desc achieves the highest AUC of 98.66%. MDL Public Keys also outperformed the other representations for NB, with a sensitivity of 96.71%, specificity of 86.97%, and an AUC of 97.99%. Another representation that exhibits a solid performance across the whole study is ECFP_4 (which is in line with previous studies [65], [66]). This fingerprint has the best sensitivity for SVM, 99.55%, the second best AUC for RF, 99.07%, and the second best sensitivity, 97.15%, and AUC, 94.25% for NB. A conceptually related fingerprint, namely FCFP_4, shows surprisingly worse performance than MDL Public Keys and ECFP_4 fingerprints by having smaller AUC values for RF, SVM, and NB, 98.16%, 94.19%, and 80.80% respectively. Molecular descriptors, both PP_desc and Atom Counts, perform well: PP_desc reports better AUC for RF and SVM, 98.93% and 98.66% respectively, than FCFP_4, 98.13% and 94.19% respectively. Atom Counts descriptors also outperform FCFP_4 in SVM in terms of AUC, 98.02% the former and 94.19% the latter. On the other hand, PP_desc and Atom Counts underperformed when used with NB, where the AUC obtained was 61.57% and 58.95%, respectively.

**Table 3:** Classification results of the test set.

	Random Forest			SVM			Naïve Bayes			Average		
	Sensitivity	Specificity	AUC	Sensitivity	Specificity	AUC	Sensitivity	Specificity	AUC	Sensitivity	Specificity	AUC
**PP_desc**	99.17%	88.60%	98.93%	96.82%	88.93%	98.66%	42.51%	86.56%	61.57%	79.50%	88.03%	86.39%
**Atom Counts**	97.91%	85.57%	97.33%	98.05%	84.10%	98.02%	36.66%	92.90%	58.95%	77.54%	87.52%	84.77%
**ECFP4**	99.80%	86.27%	99.07%	99.55%	83.43%	98.23%	97.15%	83.29%	94.25%	98.83%	84.33%	97.18%
**FCFP4**	99.55%	87.84%	98.16%	81.89%	86.53%	94.19%	99.75%	44.80%	80.80%	93.73%	73.06%	91.05%
**MDL**	99.84%	88.79%	99.13%	98.54%	86.48%	97.45%	96.71%	86.97%	97.99%	98.36%	87.41%	98.19%
**Average**	99.26%	87.41%	98.52%	94.97%	85.90%	97.31%	74.56%	78.90%	78.71%	89.59%	84.07%	91.52%

Results for the test set, including the percentage of correctly classified metabolites (Sensitivity), the percentage of correctly classified non-metabolites (Specificity) and the Area Under the Curve (AUC). It can be observed that the best combination of descriptor and classifier is MDL Public Keys and Random Forest and that the second best is ECFP_4 fingerprints and Random Forest. Interestingly, physicochemical descriptors (PP_desc) perform well both with Random Forest and Support Vector Machines classifiers. (A molecule is considered metabolite if its metabolite-likeness >50%).

By looking at the average AUC results for the different representations we conclude that MDL Public Keys (with 98.19%) and ECFP_4 (with 97.18%) are the best performing representations overall. If we observe the average results obtained by the classifiers, RF outperforms SVM and NB in each category with averages of 99.26% sensitivity, 87.41% specificity, and 98.52% AUC.

From the results presented in this work we see that with the optimal combination of molecular descriptors and classifier, MDL Public Keys and RF, 99.84% of the metabolites and 88.79% of the non-metabolites in the test set are classified correctly. These results are slightly better than those presented by Gupta et al. [28], who reported 97% correct predictions for KEGG metabolites using RF and global molecular descriptors, which are similar to the PP_desc descriptors used in the current work. While these 97% correct predictions were achieved on the dataset used to train the model, our 99.84% correctly classified metabolites were not employed in training the model. Interestingly, it is also observed in our predictions that metabolites have a smaller false positive rate than non-metabolites, which reinforces the idea that it is easier to determine *what makes a metabolite a metabolite*, than what makes a non-metabolite a non-metabolite. The ZINC molecules that have been classified as metabolites (some of them shown in Figure S4), form an interesting set for further research, since according to the models they exhibit metabolite-like features, which would give them an increased likelihood of being bioactive in experimental screening [64].

With respect to the classification algorithms, RF and SVM have demonstrated their status as the ‘state of the art’ in machine learning, as applied to this dataset. This good performance comes however at the expense of having to optimize metaparameters, which is more demanding for SVM, where finding the right gamma and cost results in changing the value ranges multiple times. From this experience, when facing a classification problem where objects are described by a large number of variables and only a modest computational power is available, RF is a good compromise.

As seen in previous research, ECFP_4 is a solid ‘all-round performer’ [65], [66], which obtains good results in combination with the different classification approaches. The most surprising feature is that with simpler molecular representations than ECFP_4, like MDL Public Keys or PP_desc molecular descriptors, one can achieve similar or slightly improved results from the above, as it has been observed before [67]. This finding confirms the idea that (at least known) ‘Metabolite Space’ is a well-defined subset of all ‘Chemical Space’, and that hence its diversity can be modeled with success using either 1D or 2D descriptors.

**Figure 6:**
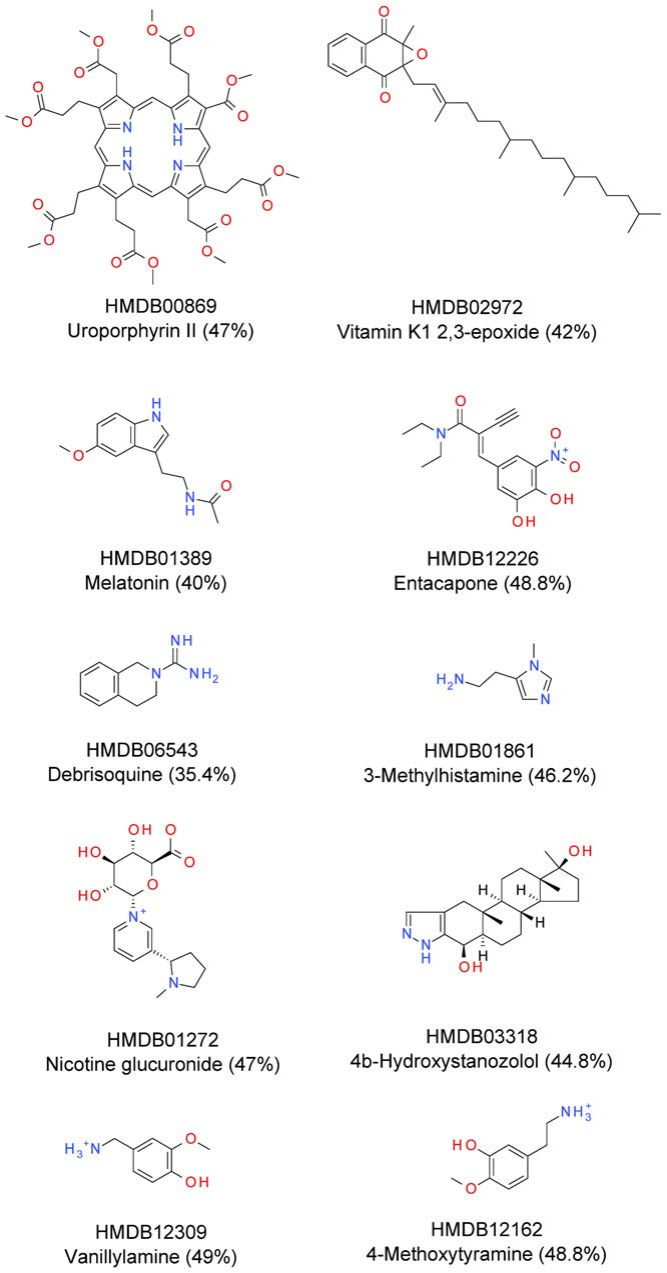
Metabolites in the test set predicted as non-metabolites. The 10 only false negative metabolites from the test set. These metabolites obtained a Metabolite-likeness score smaller than 50%, therefore being classified as non-metabolites, using the best model, MDL Public Keys and Random Forest. Debrisoquine obtains the lowest score; it is a drug that was not taxonomically described as such. 9 out of 10 compounds have 40% or more metabolite-likeness, which is very close to our cut-off used to predict metabolites.

Apart from the discussion of general model performance we also investigated cases where our model failed, which may be either due to wrong data annotation or wrong predictions of the model. Figure 6 depicts false negative predictions, *i.e.* those metabolites with a metabolite-likeness value of 50% or lower, and which were therefore being considered as non-metabolites in combination with the MDL Public Keys and the RF classification method. Although these molecules would be considered non-metabolites by our model, 9 out of 10 obtain a metabolite-likeness of 40% or more. It is interesting to note that the lowest scoring compound, debrisoquine with a score of 35.4%, is in fact a drug. Since it was not described as such by the HMDB taxonomy, our filtering step did not eliminate it. The same occurs for entacapone, which is a drug and has a predicted metabolite-likeness of 48.8%. Nevertheless, our classification method was able to assign to both drugs the lowest metabolite-likeness scores. Non-endogenous compounds are also present in this group of compounds, such as nicotine glucuronide, and 4b-Hydroxystanozolol, a metabolite of the synthetic anabolic steroid stanozolol. In the same fashion, we find in this set vanillylamine, with 49% of predicted metabolite-likeness, which is a metabolite of the natural product Vanillin and which structure resembles the endogenous metabolite 4-Methoxytyramine, which obtains a metabolite-likeness score of 48.8%. Unfortunately, some endogenous metabolites like Uroporphyrin II, 3-Methylhistamine, Melatonin, and Vitamin K1 2,3-epoxide, received a low score without an obvious reason, and they are hence false-negative predictions of our model.

### Prospective Validation

**Figure 7:**
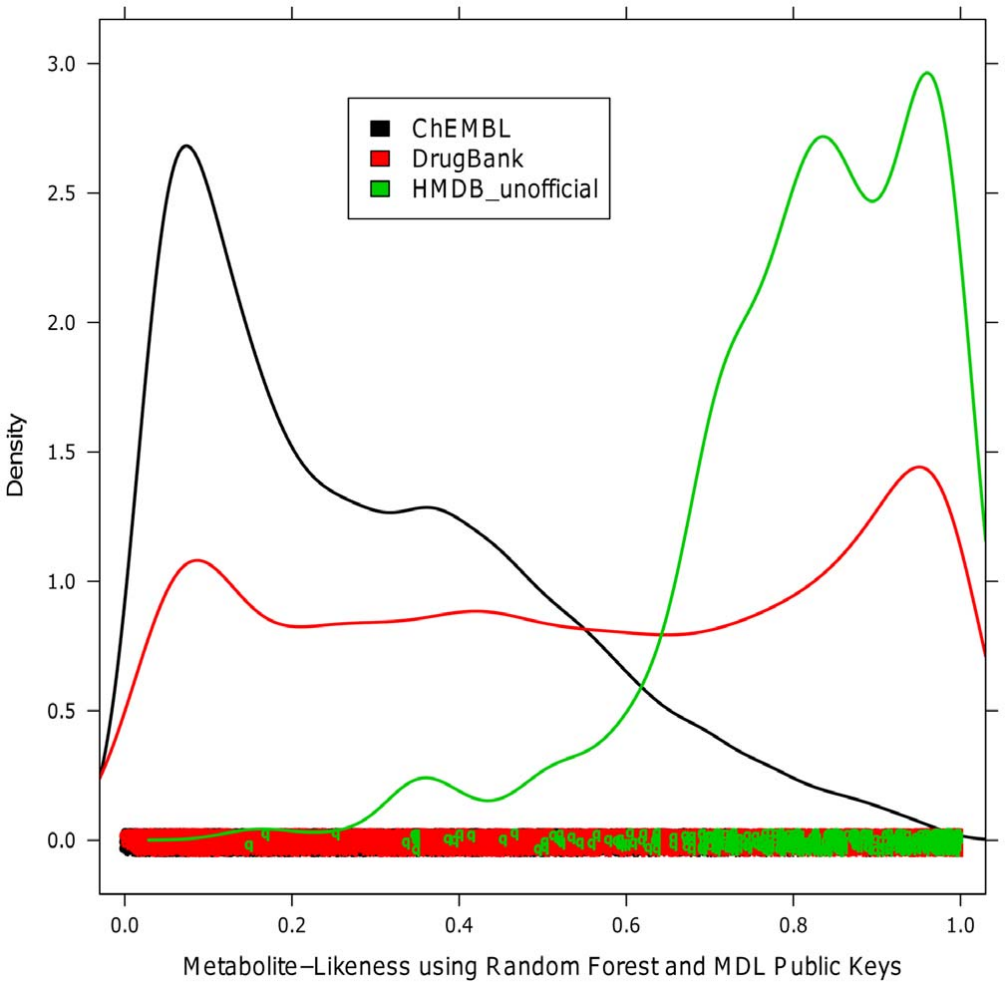
Metabolite-likeness distribution of the prospective validation sets. Distribution of predicted metabolite-likeness for the three classes of molecules in the prospective evaluation set using our best predicting model, RF and MDL Public Keys (namely metabolites from HMDB, drugs from DrugBank and bioactive compounds from ChEMBL). Most of the metabolites are predicted at a metabolite-likeness of 60% or higher. Most of non-metabolites from ChEMBL obtain low values. Drugs from DrugBank are spread across the whole range of values, with higher concentrations at both ends, which indicate a presence of synthetic drugs, for low values, and metabolite-like drugs at high values.

Three prospective datasets containing metabolites, drugs, and small molecules, were next classified using our two best performing models, using RF and either MDL Public Keys or PP_desc. The results are displayed in Table 4 and indicate that 95.84% of the new metabolites (obtained after model training has been finished) are correctly classified as metabolites, indicating the generalizability of our model to classify new data. As for the drugs (represented by DrugBank compounds), 54.37% are assigned a metabolite-likeness of 50% or higher, which is in accordance with our assumption that many drugs indeed resemble metabolites (as has been presented before [64]). For the third dataset, the screening compounds from ChEMBL, molecules predicted to be metabolites only represent 22.39% of the total dataset, hence a smaller percentage than for drugs. In Figure 7 the distributions of metabolite-likeness for each dataset are visualized. We see that most of the new HMDB compounds (HMDB_unofficial) show high values of metabolite-likeness, while the ChEMBL molecules give values that are accumulating at the lower-scoring end of the distribution. The DrugBank molecules on the other hand are evenly distributed among all the metabolite-likeness ranges, with slight peaks at both the metabolite-like, as well as the non-metabolite-like end of the spectrum. This result is in accordance to the work of Ertl et al. [68], where a Natural Product-Likeness score was reported after studying natural products, drugs, and screening compounds. Natural products are molecules produced by living organisms, and therefore they can be regarded as to some extend similar to the human metabolites we employed in our work. Ertl et al. concluded that drugs are more similar to natural products than screening compounds, a similar finding to what we have presented. This biogenic bias is also present in screening libraries, as presented by Hert et al. [64]; however, the wide spread of drugs along the spectrum of metabolite-likeness (in particular with slight peaks at either end of the scale) has not been previously reported.

**Table 4:** Percentage of molecules classified as metabolites or non-metabolites for three independent sets.

	RF Prediction	
	Metabolites	Non-Metabolites
**HMDB_unofficial**	95.84%	4.15%
**DrugBank**	54.37%	45.62%
**ChEMBL**	22.39%	77.61%

95.84% of independent metabolites are correctly classified. More than half of the drugs in DrugBank are considered metabolites. Only 22.39% of the screening compounds in ChEMBL are predicted as metabolites. (A molecule is considered metabolite if its metabolite-likeness >50%.).

**Figure 8:**
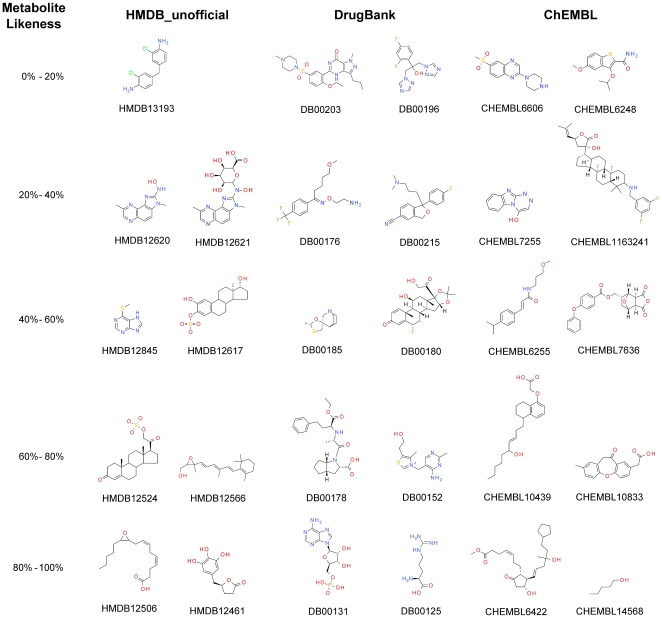
Molecules of the prospective validation sets with different predicted metabolite-likeness values. Compounds of the 3 classes present in the prospective evaluation set using our best predicting model, RF and MDL Public Keys, sorted according to their predicted metabolite-likeness. Non-metabolite compounds exhibit moieties characteristic of metabolites like carboxylic acids and phosphate groups, which make them obtain high values of metabolite-likeness.

While numerical performance is one thing, the chemical interpretation of model predictions remains crucial. Hence, in order to explore further the results of the prospective validation, molecules of the three different classes (metabolites, drugs, bioactive compounds), which fall into different bins of metabolite-likeness scores, are presented in Figure 8. The first noticeable feature is the absence of a metabolite with a predicted metabolite-likeness smaller than 10%, underlining the homogeneity of metabolites as a class (as opposed to non-metabolites). As a matter of fact, the metabolite HMDB13193 obtained the lowest metabolite-likeness, 17%, contains two chlorine atoms, which is not common in metabolites. Another interesting situation occurs with molecules that have a steroid scaffold, a common fragment in endogenous metabolites. Metabolite HMDB12524 and drug DB00180 (flunisolide) obtain metabolite-likeness values of 60.6% and 52%, respectively. Here flunisolide possesses a fluorine atom, which is not frequent in metabolites, and which might have hence reduced its metabolite-likeness score. Conversely, ChEMBL compound CHEMBL1163241 also has the steroid scaffold but obtains a score of just 35.2% on the metabolite-likeness scale, corresponding related to having two fluorine atoms and a secondary amine, features that the classification tree revealed to be common in non-metabolites. Finally, examples of compounds with high values of predicted metabolite-likeness are DB00131 (adenosine monophosphate), DB00125 (L-arginine), CHEMBL6422, and CHEMBL14568, which receive 84.2%, 99%, 82.8%, and 96.8% respectively. Adenosine monophosphate includes the phosphate group, frequently found in metabolites together with two hydroxyl groups. Metabolite-likeness features of L-Arginine, like linearity and a carboxylic group, outweigh the non-metabolite features like the nitrogen containing functional groups. Compound CHEMBL6422 possesses a carboxylic acid and hydroxyl functionalities, while and CHEMBL14568 is small, linear, and also exhibits a hydroxyl group, leading to a very high metabolite-likeness score.

The results obtained from the prospective validation demonstrate that our model is successful at identifying whether a molecule is a metabolite or not, which we expect to help studies that involve metabolite identification in the future. Furthermore, metabolite-likeness helps to detect non-metabolites that exhibit features characteristic of metabolites, which can be of interest for drug discovery In our future work, we will explore both of those avenues with results to be communicated shortly.

In this work we evaluated various machine-learning models with respect to their ability to discriminate metabolites from non-metabolites, and hence, to calculate the metabolite-likeness score of a given molecule. Our best model detects 99.84% of the metabolites from the test set and 95.84% of the metabolites from a prospective validation set, hence underlining the applicability of the classifier to the majority of novel metabolites. While we confirm that drugs are, on average, more metabolite-like than other compound classes, we noted a considerable spread of drugs across the metabolite-likeness spectrum, with two small (but distinct) peaks at either end of the spectrum, illustrating that both synthetic molecules and metabolite-like compounds may become successful drugs. As for the application side, metabolite-likeness is a tool to rank compounds that ‘need’ to resemble metabolites, which may be (as above) certain types of drugs, but also in particular candidate structures in metabolite identification. Given the performance of our model, we will now continue with our work to apply our model in precisely those areas. Accordingly, we expect to use this tool in metabolomics studies where no database match is found for the unknown compound and therefore, candidate structures are generated based on mass spectrometry data, e.g. elemental composition, using a structure generation tool. These output molecules would be then ranked according to their Metabolite-Likeness. Furthermore, we have also studied which functional groups, fragments, and physicochemical properties help describe the Metabolite Space. Our findings give a general idea of what metabolites look like, but also encourage us to look closer at the different subclasses of metabolites and to explore the applicability of a local model approach if we want to expand our knowledge of metabolites.

## Supporting Information

Figure S1 **PCA of the PP_desc and MDL Public Keys that the RF model considers important.** The importance criterion is the Mean Decrease Accuracy. The separation of both classes is slightly improved for PP_desc using these important variables if compared with the PCA score plot in Figure 1.
(TIF)

Figure S2 **PCA of the PP_desc and MDL Public Keys that the RF model considers important.** The importance criterion is the Mean Decrease Gini. The separation of both classes is slightly improved for PP_desc using these important variables if compared with the PCA score plot in Figure 1.
(TIF)

Figure S3 **Hierarchical clustering of PP_desc and MDL Public Keys.** Plots of the first two dimensions of the Hierarchical Clustering. For PP_desc: A, using all variables; B, using the important variables according to Accuracy decrease; C, using the important variables according to Gini decrease. For MDL Public Keys: A, using all variables; B, using the important variables according to Accuracy decrease; C, using the important variables according to Gini decrease. In all cases the optimal cut of the dendogram, according to the maximum loss of inertia, returns 3 clusters.
(TIF)

Figure S4 **Non-metabolites predicted as metabolites.** Some non-metabolites from the test set that obtained a Metabolite-likeness score greater than 50%, therefore being classified as metabolites, using the best model, MDL Public Keys and Random Forest. These are the 20 cluster centers selected from the clustering performed on all the false positives.
(TIF)

Table S1 Optimal metaparameters for classifiers. mtry for Random Forest, Gamma and Cost for Support Vector Machines, obtained after performing Cross Validation on the training set.
(DOC)

Table S2 Importance given to the PP_desc descriptors by Random Forest. High values on Mean Decrease Accuracy and in Mean Decrease Gini indicate that this variable is important to discern between metabolites and non-metabolites. These importance values have been obtained from the Random Forest model built with the training set.
(DOC)

Table S3 Importance given to the MDL Public Keys by Random Forest. High values on Mean Decrease Accuracy and in Mean Decrease Gini indicate that this variable is important to discern between metabolites and non-metabolites. These importance values have been obtained from the Random Forest model built with the training set.
(DOC)

Table S4 Cumulative percentage of variance explained of the first 8 principal components. PCA was performed on the Atom Counts, PP_desc, and MDL Public Keys datasets.
(DOC)
