# Supplementary figures and images for: Understanding and Classifying Metabolite Space and Metabolite-Likeness
Source: PLoS One. 2011 Dec 14;6(12):e28966. doi: 10.1371/journal.pone.0028966 (PMC3237584; doi:10.1371/journal.pone.0028966)

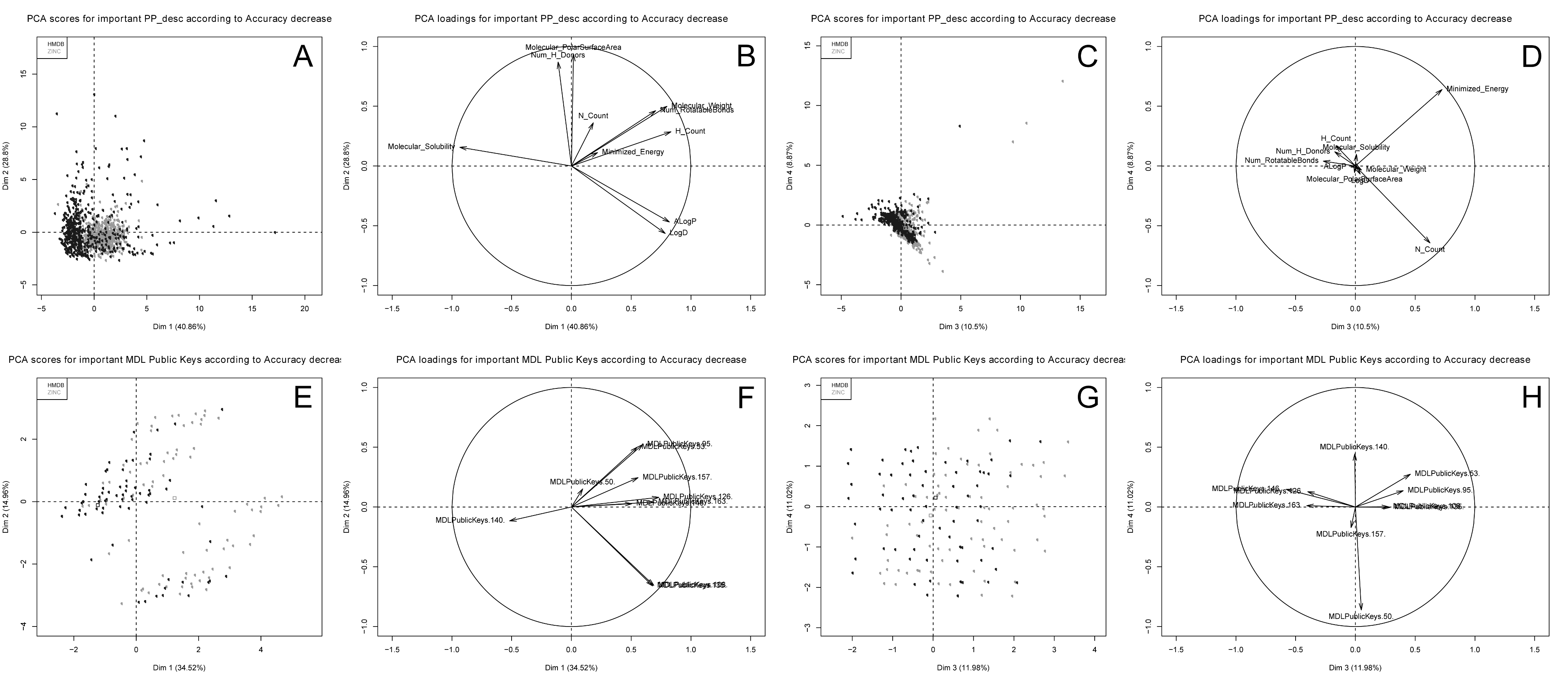

Supplement: Figure S1 — PCA of the PP_desc and MDL Public Keys that the RF model considers important. The importance criterion is the Mean Decrease Accuracy. The separation of both classes is slightly improved for PP_desc using these important variables if compared with the PCA score plot in Figure 1. (TIF) [file pone.0028966.s001.tif]

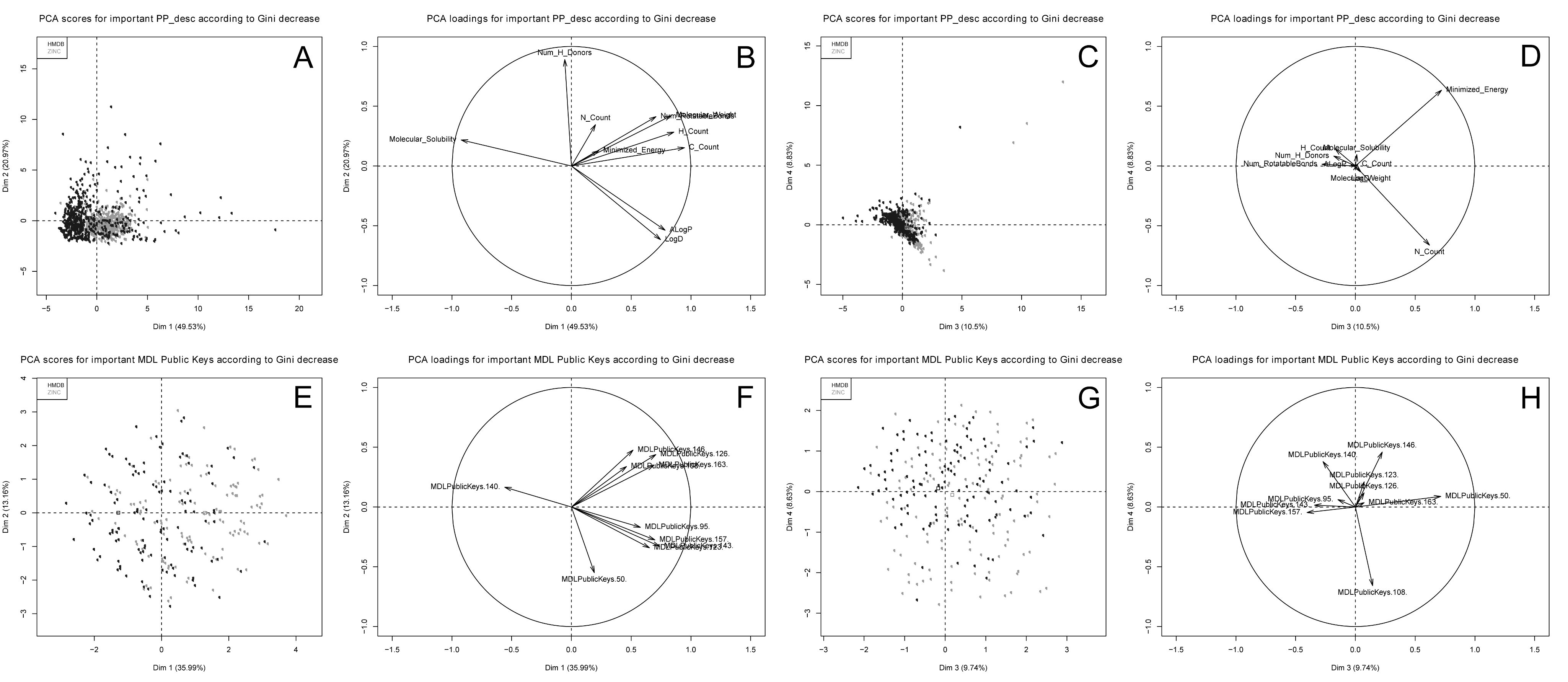

Supplement: Figure S2 — PCA of the PP_desc and MDL Public Keys that the RF model considers important. The importance criterion is the Mean Decrease Gini. The separation of both classes is slightly improved for PP_desc using these important variables if compared with the PCA score plot in Figure 1. (TIF) [file pone.0028966.s002.tif]

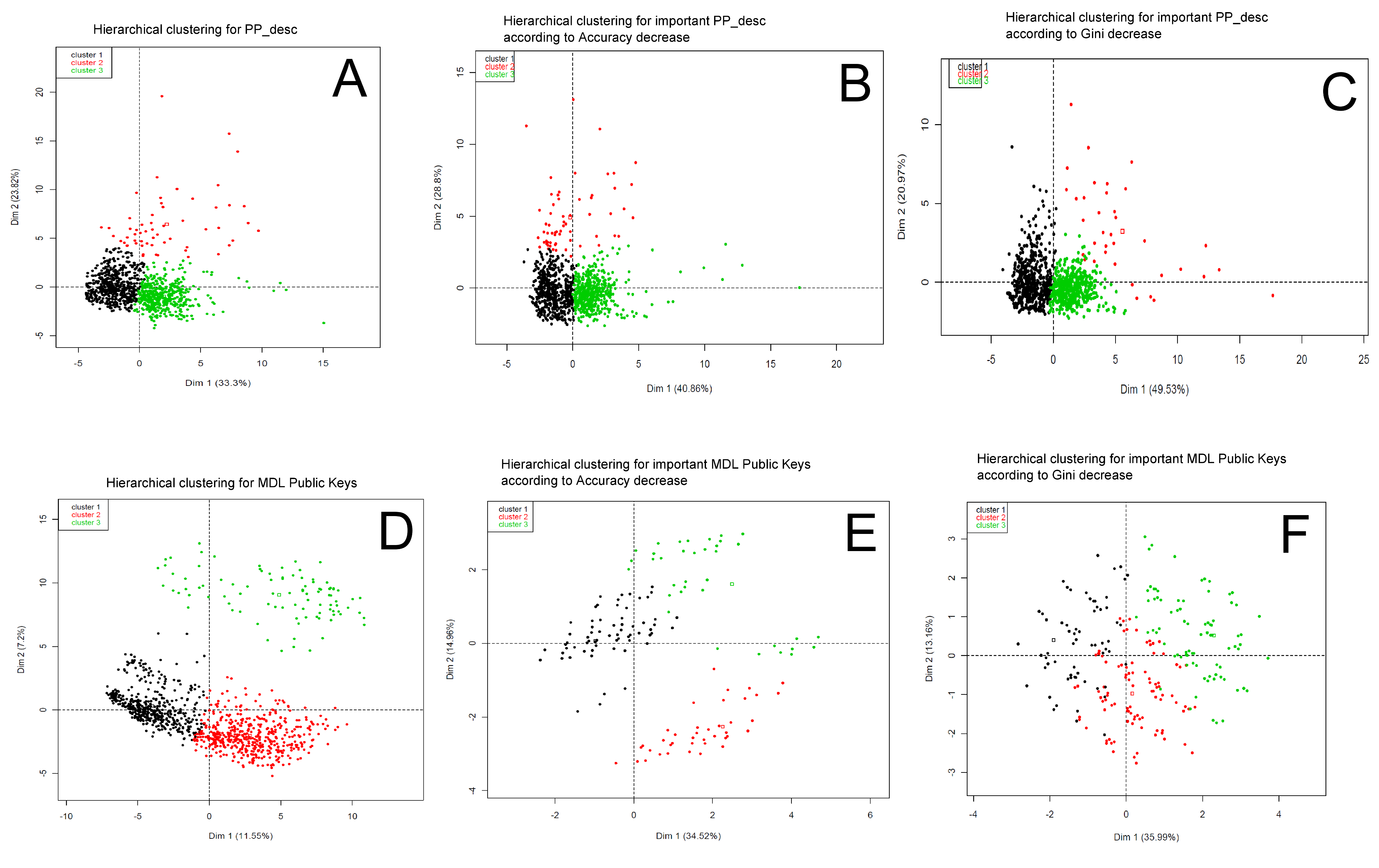

Supplement: Figure S3 — Hierarchical clustering of PP_desc and MDL Public Keys. Plots of the first two dimensions of the Hierarchical Clustering. For PP_desc: A, using all variables; B, using the important variables according to Accuracy decrease; C, using the important variables according to Gini decrease. For MDL Public Keys: A, using all variables; B, using the important variables according to Accuracy decrease; C, using the important variables according to Gini decrease. In all cases the optimal cut of the dendogram, according to the maximum loss of inertia, returns 3 clusters. (TIF) [file pone.0028966.s003.tif]

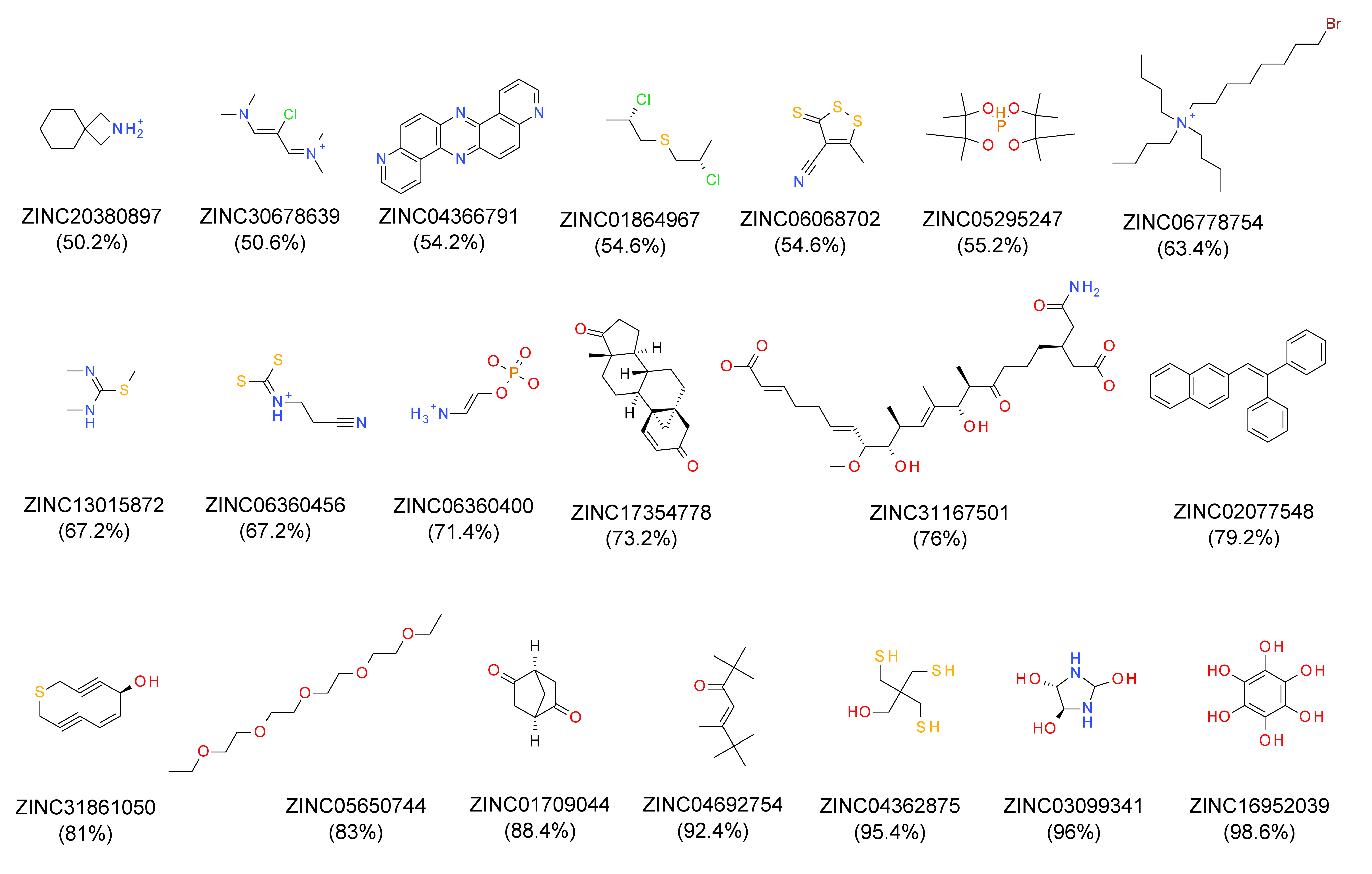

Supplement: Figure S4 — Non-metabolites predicted as metabolites. Some non-metabolites from the test set that obtained a Metabolite-likeness score greater than 50%, therefore being classified as metabolites, using the best model, MDL Public Keys and Random Forest. These are the 20 cluster centers selected from the clustering performed on all the false positives. (TIF) [file pone.0028966.s004.tif]
