# Supplementary material for: Understanding and Classifying Metabolite Space and Metabolite-Likeness
Source: PLoS One. 2011 Dec 14;6(12):e28966. doi: 10.1371/journal.pone.0028966 (PMC3237584; doi:10.1371/journal.pone.0028966)
Supplement: Table S1 — Optimal metaparameters for classifiers. mtry for Random Forest, Gamma and Cost for Support Vector Machines, obtained after performing Cross Validation on the training set. (DOC) [file pone.0028966.s005.doc]

|  | **mtry** | **Gamma** | **Cost** |
| --- | --- | --- | --- |
| **PP_desc** | 9 | 4.768372e-07 | 32768 |
| **Atom Counts** | 2 | 4.882812e-04 | 262144 |
| **ECFP4** | 10 | 3.051758e-05 | 16 |
| **FCFP4** | 30 | 1.907349e-06 | 8192 |
| **MDL** | 30 | 1.907349e-06 | 8192 |
