# Supplementary material for: Understanding and Classifying Metabolite Space and Metabolite-Likeness
Source: PLoS One. 2011 Dec 14;6(12):e28966. doi: 10.1371/journal.pone.0028966 (PMC3237584; doi:10.1371/journal.pone.0028966)
Supplement: Table S2 — Importance given to the PP_desc descriptors by Random Forest. High values on Mean Decrease Accuracy and in Mean Decrease Gini indicate that this variable is important to discern between metabolites and non-metabolites. These importance values have been obtained from the Random Forest model built with the training set. (DOC) [file pone.0028966.s006.doc]

|  | **HMDB** | **ZINC** | **MeanDecreaseAccuracy** | **MeanDecreaseGini** |
| --- | --- | --- | --- | --- |
| **N_Count** | 1.3067688 | 1.2609013 | 1.0014388 | 46.898305 |
| **Molecular_Solubility** | 1.2788442 | 1.02582 | 0.9656276 | 96.271476 |
| **LogD** | 1.2191826 | 1.0680953 | 0.9642439 | 50.24542 |
| **Num_H_Donors** | 1.3046778 | 0.8545617 | 0.9567927 | 29.876567 |
| **Num_RotatableBonds** | 1.012057 | 1.17285022 | 0.8988967 | 26.85855 |
| **Molecular_Weight** | 1.1004754 | 0.99654993 | 0.8885767 | 31.778377 |
| **Minimized_Energy** | 1.1796855 | 0.69780848 | 0.8681832 | 32.409426 |
| **H_Count** | 1.2011563 | 0.24332713 | 0.8579638 | 24.73855 |
| **Molecular_PolarSurfaceArea** | 1.1207631 | 0.70494526 | 0.8549427 | 23.351115 |
| **ALogP** | 0.9248217 | 0.72155958 | 0.7985026 | 25.724204 |
| **Num_Atoms** | 0.7790244 | 0.76550378 | 0.7354873 | 20.597452 |
| **Num_H_Acceptors** | 0.9853438 | 0.35682066 | 0.7293065 | 10.555502 |
| **F_Count** | 1.0559811 | -0.08105052 | 0.7200118 | 5.685859 |
| **Num_Rings** | 0.8475657 | 0.73452855 | 0.7184914 | 24.290726 |
| **C_Count** | 0.9386965 | 0.62885999 | 0.7131295 | 41.595612 |
| **Num_AromaticRings** | 0.7248383 | 0.81406126 | 0.6912011 | 22.524017 |
| **O_Count** | 0.8782411 | 0.45284146 | 0.6735855 | 10.741794 |
| **S_Count** | 0.7923043 | 0.02593264 | 0.4822221 | 4.077548 |
| **Cl_Count** | 0.7362878 | -0.37414425 | 0.3897458 | 2.297109 |
| **P_Count** | -0.133703 | 0.35386364 | 0.1807093 | 0.980051 |
| **N_Count** | 1.3067688 | 1.2609013 | 1.0014388 | 46.898305 |
