# Supplementary material for: Understanding and Classifying Metabolite Space and Metabolite-Likeness
Source: PLoS One. 2011 Dec 14;6(12):e28966. doi: 10.1371/journal.pone.0028966 (PMC3237584; doi:10.1371/journal.pone.0028966)
Supplement: Table S3 — Importance given to the MDL Public Keys by Random Forest. High values on Mean Decrease Accuracy and in Mean Decrease Gini indicate that this variable is important to discern between metabolites and non-metabolites. These importance values have been obtained from the Random Forest model built with the training set. (DOC) [file pone.0028966.s007.doc]

|  | **HMDB** | **ZINC** | **MeanDecreaseAccuracy** | **MeanDecreaseGini** |
| --- | --- | --- | --- | --- |
| **MDLPublicKeys.140** | 1.24138956 | 1.229133216 | 0.941920823 | 53.91841097 |
| **MDLPublicKeys.126** | 0.83892897 | 0.70907696 | 0.663644388 | 23.62313439 |
| **MDLPublicKeys.163** | 0.78447704 | 0.746221036 | 0.640603474 | 23.23559085 |
| **MDLPublicKeys.50** | 1.17048163 | 1.023474316 | 0.885259239 | 15.04553608 |
| **MDLPublicKeys.143** | 0.69289234 | 0.696664822 | 0.593501328 | 14.91132605 |
| **MDLPublicKeys.108** | 0.99957857 | 0.489609227 | 0.713694188 | 14.48014674 |
| **MDLPublicKeys.157** | 0.63767173 | 0.675822066 | 0.603715398 | 13.07839857 |
| **MDLPublicKeys.123** | 0.58418556 | 0.407174217 | 0.474627005 | 13.02194646 |
| **MDLPublicKeys.146** | 0.87558184 | 0.687488161 | 0.703815276 | 12.6453885 |
| **MDLPublicKeys.95** | 0.79652399 | 0.624375878 | 0.637617052 | 10.20590729 |
| **MDLPublicKeys.135** | 0.90220951 | 0.439277352 | 0.637256942 | 9.92262443 |
| **MDLPublicKeys.145** | 0.62671793 | 0.411886181 | 0.50576454 | 8.80818597 |
| **MDLPublicKeys.122** | 0.56884211 | 0.176402217 | 0.42077643 | 8.25408405 |
| **MDLPublicKeys.132** | 0.70757544 | 0.285691095 | 0.519554788 | 6.57286614 |
| **MDLPublicKeys.138** | 0.5532357 | 0.466305805 | 0.478651557 | 6.54776274 |
| **MDLPublicKeys.128** | 0.57303747 | 0.41742079 | 0.494394286 | 6.48147908 |
| **MDLPublicKeys.53** | 0.8537977 | 0.309679345 | 0.609916532 | 6.05340169 |
| **MDLPublicKeys.82** | 0.72725636 | 0.393078873 | 0.547102936 | 5.88552798 |
| **MDLPublicKeys.76** | 0.46225838 | 0.21721732 | 0.368584167 | 5.84646577 |
| **MDLPublicKeys.140** | 1.24138956 | 1.229133216 | 0.941920823 | 53.91841097 |
| **MDLPublicKeys.126** | 0.83892897 | 0.70907696 | 0.663644388 | 23.62313439 |
