# Supplementary material for: Understanding and Classifying Metabolite Space and Metabolite-Likeness
Source: PLoS One. 2011 Dec 14;6(12):e28966. doi: 10.1371/journal.pone.0028966 (PMC3237584; doi:10.1371/journal.pone.0028966)
Supplement: Table S4 — Cumulative percentage of variance explained of the first 8 principal components. PCA was performed on the Atom Counts, PP_desc, and MDL Public Keys datasets. (DOC) [file pone.0028966.s008.doc]

| **Component** | **Atom Counts** | **PP_desc** | **MDL Public Keys** |
| --- | --- | --- | --- |
| 1 | 25.44165 | 33.29614 | 11.54531 |
| 2 | 44.54251 | 57.11561 | 18.74551 |
| 3 | 58.67426 | 66.05534 | 23.8788 |
| 4 | 71.17018 | 71.99137 | 28.55269 |
| 5 | 82.20865 | 77.25728 | 32.71371 |
| 6 | 92.29973 | 82.01365 | 36.04002 |
| 7 | 98.71144 | 86.32577 | 38.95153 |
| 8 | 100 | 90.20601 | 41.51726 |
